# The Effect of Monochromatic Infrared Photo Energy on the Irritability of Myofascial Trigger Spot of Rabbit Skeletal Muscle

**DOI:** 10.1155/2015/816956

**Published:** 2015-09-09

**Authors:** Ta-Shen Kuan, Yu-Ching Lin, Wei-Chih Lien, Pei-Chun Hsieh, Yu-Ting Chung, Sheng-Hsiang Lin, Li-Wei Chou

**Affiliations:** ^1^Department of Physical Medicine and Rehabilitation, College of Medicine, National Cheng Kung University, Tainan 70101, Taiwan; ^2^Department of Physical Medicine and Rehabilitation, National Cheng Kung University Hospital, College of Medicine, National Cheng Kung University, Tainan 70403, Taiwan; ^3^Institute of Clinical Medicine, College of Medicine, National Cheng Kung University, Tainan 70101, Taiwan; ^4^School of Chinese Medicine, College of Chinese Medicine, China Medical University, Taichung 40402, Taiwan; ^5^Department of Physical Medicine and Rehabilitation, China Medical University Hospital, Taichung 40447, Taiwan

## Abstract

*Objective. *To determine whether the vasodilatation effect of monochromatic infrared photo energy (MIRE) had the potential for the treatment of myofascial trigger spot (MTrS) in rabbits. *Design*. A randomized-controlled animal study. *Subjects.* Twelve adult New Zealand rabbits. *Methods.* For each rabbit, a MTrS (equivalent to a myofascial trigger point in humans) in one side of the biceps femoris muscle was randomly selected for MIRE treatment (experimental side), while another MTrS in the other side (control side) received a sham treatment. The intervention consisted of a daily 40 minutes treatment, three times per week for 2 weeks. The prevalence of endplate noise (EPN) loci in the MTrS was assessed before, immediately after, and one week after the completion of the 2-week treatment. *Results.* MIRE could suppress the prevalence of EPN in the MTrS. The degree of reduction in EPN prevalence in the MTrS between the experimental side and the control side was significantly different immediately after MIRE treatment, but not significantly different one week after MIRE treatment. *Conclusion.* Our study suggests that MIRE may be a useful therapeutic option for the management of the myofascial trigger point in humans.

## 1. Introduction

Monochromatic infrared photo energy (MIRE) is a type of photobiomodulation. MIRE has been proposed as a therapeutic modality for several clinical conditions such as peripheral neuropathy, wound healing, and pain management [1]. The device delivering MIRE, known as the Anodyne Therapy System (ATS), has been approved by the FDA for temporarily increasing local blood circulation and reducing pain [1]. Several recent studies of MIRE focused on the restoration of sensation and wound healing in patients with diabetic peripheral neuropathy [2–6]. However, the effect of pain reduction by MIRE on a common muscle pain condition, that is, myofascial pain syndrome (MPS), has not been fully studied.

MPS is characterized by the presence of a myofascial trigger point (MTrP), a localized hyperirritable spot in a palpable taut band of skeletal muscle fibers [7–10]. An animal model using rabbits has been developed and found to be very useful for studying the pathophysiology of the MTrP [11, 12]. In the adult rabbit skeletal muscle, the taut bands are similar to that in the human muscle and can be identified by finger palpation. When a certain sensitive site in the palpable taut band is squeezed or compressed by a finger, the rabbit would express as if it suffers pain or discomfort (such as screaming, kicking, or withdrawing). The expression is seldom observed when areas outside the sensitive site are compressed. This hypersensitive site is defined as a myofascial trigger spot (MTrS), which is in many ways similar to the MTrP in human muscle [11, 12].

Rabbit localized twitch responses, which are similar to human local twitch responses (LTRs) as one characteristic of the MTrP, can be elicited much easier at the MTrS than at other sites in the same muscle [11, 12]. Another characteristic of the MTrP is the electromyographic (EMG) activity recorded in the MTrP region, defined as “spontaneous electrical activity (SEA)” by Simons et al. [13]. SEA consisted of two components: the endplate noise (EPN) and the endplate spike. EPN is recorded much more often in an MTrP region than in a non-MTrP region [13]. The minute locus from which SEA can be recorded has been defined as an* active locus *of an MTrP [14]. Hong proposed a “Multiple Loci Theory” (many sensitive loci in an MTrP region), and many LTRs can be elicited and many SEAs can be recorded in an MTrP region [7, 8]. It has been shown that the prevalence of EPN in an MTrP region is highly correlated with the irritability of that MTrP [15]. We hypothesize that MIRE is effective for the management of MPS through the vasodilatation effect of MIRE to improve the local microcirculation. The objective of this study is to test if MIRE can significantly decrease the prevalence of EPN in an MTrS in the rabbit skeletal muscle.

## 2. Material and Methods

### 2.1. General Design

**Figure 1:**
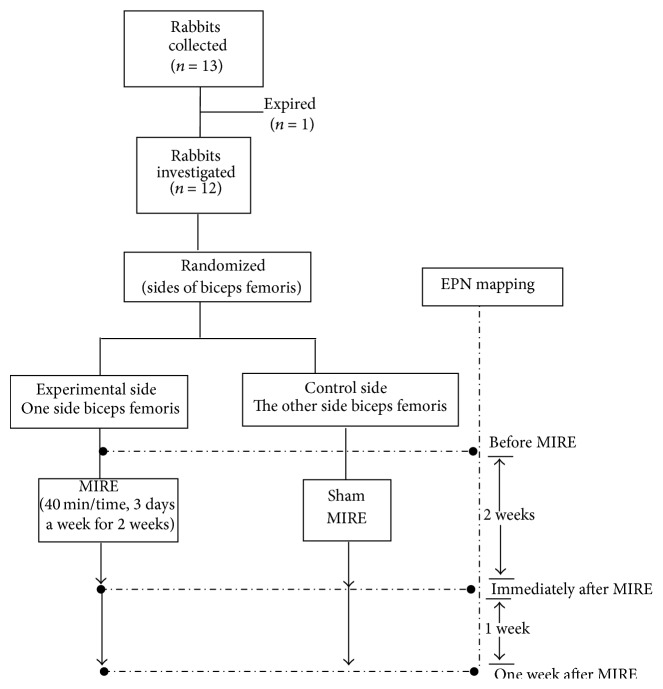
Diagram of the experimental protocol. EPN: endplate noise. MIRE: monochromatic infrared photo energy.

This was a randomized-controlled experiment and was approved by the Institutional Animal Care and Use Committee of National Cheng Kung University. The procedures consisted of the application of MIRE over the MTrS region of the rabbit biceps femoris muscle in one randomly selected side (experimental side) and a sham treatment in the other side (control sides). For sham MIRE, the diodes were inactivated so that no infrared photo energy was emitted. The prevalence of EPN in the MTrS region (EPN mapping) in both the experimental and the control sides was measured before, immediately after, and one week after the completion of the whole course (2-week period) of MIRE treatment (Figure 1).

### 2.2. Animals

Thirteen adult New Zealand rabbits (3~5 Kg) were collected but one was expired before the start of the treatment. For the remaining 12 rabbits, each was kept alone in a large cage, with a 12-hour alternating light-dark cycle, sawdust bedding, and free access to food and water. Before anesthesia, one investigator palpated the rabbit's biceps femoris muscle in both sides with his finger to locate the most tender spot (MTrS) in the taut band. The MTrS was marked, and the rabbit was then anesthetized with an intramuscular injection of Ketamine 0.05 mg/GBW [16] over its paraspinal muscle. The skin across the lateral aspect of the posterior thigh was shaved bilaterally and the rabbit was placed on a thermostatically controlled circulating-water heating pad with the temperature adjusted to 37°C. Subsequent intravenous injections of Pentothal at 0.01 g/mL were given every 20–30 minutes to maintain the anesthetic level so that most of the spinal reflexes were preserved. Respiration and rectal temperature were checked every 15 minutes, and heart rate and oxygen saturation were monitored every 5 minutes.

Instead of fully exploring the biceps femoris muscle with incision of the entire skin, which often resulted in massive bleeding and confounding the vasodilatation effect of MIRE, only a small longitudinal incision (about 3~4 cm) was made over the skin about 1 cm beside the MTrS that allowed the insertion of EMG needle.

### 2.3. Assessment of EPN Prevalence

A 4-channel NICOLET Viking IV EMG unit was used for the assessment of EPN prevalence. The high-cut frequency filter was set at 1,000 Hz and the low-cut at 100 Hz. The gain was generally set at 20 *μ*V per division for recordings. At the usual sweep speed of 10 ms per divisions, one screen presented 100 ms of record. A 37 mm, disposable, monopolar needle electrode was connected to channel 1 of the preamplifier box of the EMG unit and was used to search for the EMG activity (SEA) of an MTrS. The control needle electrode, which was connected to channel 2, was inserted into a normal muscle tissue (nontaut band, non-MTrS). A clip used for the surface electrode was attached onto the nearby skin. It was served as the common reference electrode by connecting it to both channels through a “Y” connector. The ground electrode was clipped to another site of nearby skin. Room temperature was maintained at 25 ± 1°C.

The search needle was inserted parallel to the direction of the muscle fibers into the region of an MTrS at an angle of approximately 45°~60° to the surface of the muscle. After the initial insertion to a point just short of the depth of an MTrS, the needle was advanced very slowly. Each advance was made through the least possible distance (usually 1-2 mm for one advancement) by simultaneously rotating the needle to facilitate smooth entry through the muscle tissue. Large advance was avoided because of the minute size of an SEA locus and the likelihood of inducing an rabbit-LTR instead of finding a locus of SEA. When the needle approached an SEA locus, the continuous distant electrical activity (EPN) could be heard. The needle was then pressed laterally in four directions (forward and back, right and left), one of which often resulted in appearance of EPN. If not, the needle was advanced a minimum distance, which then usually resulted in appearance of EPN. A site was an* active* locus when EPN was identified if:

1. noise-like potentials persisted continuously for more than 3 screens (300 ms);
2. the potential with an amplitude of >10 *μ*V (which was more than twice the instrumentation noise level of 4 *μ*V that was observed in control recordings taken at the beginning and upon completion of each track);
3. the adjacent control channel did not record potentials greater than instrumentation noise level.

**Figure 2:**
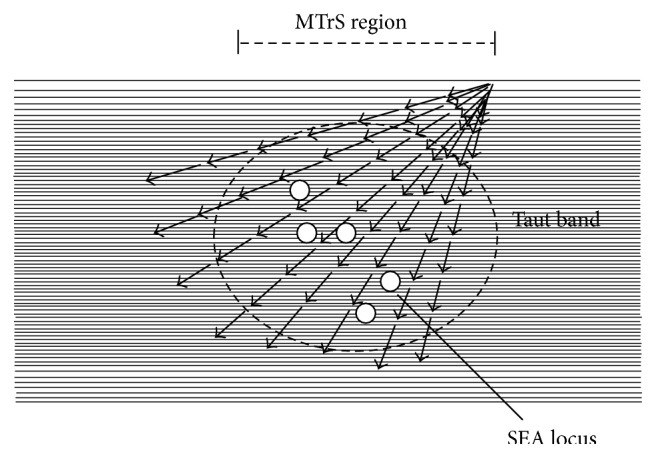
The advancement of the recording needle electrode in an MTrS region for searching for SEA locus. MTrS: myofascial trigger spot. SEA: spontaneous electrical activity.

There were 8 advancements of needling (each advancement about 1 mm) in one needling track in an MTrS region. When the recording electrode approached its end of advancement, it was withdrawn to the subcutaneous level, but not out of the skin, and then reinserted the electrode into another track (maybe 1 mm adjacent to the previous track). There were totally 8 tracks investigated in one MTrS region (Figure 2). This allowed for the exploration of 64 different loci searching for EPN in the region of one MTrS (EPN mapping). All the SEA loci found in these 64 searching loci in one MTrS region were recorded as the prevalence of EPN and were saved for later data analysis.

### 2.4. Application of MIRE

MIRE delivers a single (monochromatic) wavelength of near infrared photo energy at 890 nanometers; the machine delivering MIRE which we used in this experiment was the Anodyne Therapy System (ATS) (Anodyne Therapy LLC, Tampa, FL). The ATS consisted of a base power unit and therapy pads, which contained an array of 60 superluminous gallium aluminum arsenide diodes in each pad (3 cm × 7.5 cm, or 22.5 cm^2^). Therefore, MIRE is a kind of light emitting diode (LED). MIRE delivers pulsed adjustable radiant power of up to 10 milliwatts per diode, a power density per diode array of up to 10 milliwatts/cm^2^, and an energy density of up to 1.6 joules/cm^2^/minute [1]. However, the effectiveness of the photo energy will be influenced by many factors, such as wavelength, radiant power, pulsation, energy density, and skin contact. According to the manufacturer documentations, MIRE only warms the targeted epidermis slightly; the local heating effect may not be a serious issue. Thus the therapy pad with its diode array can be safely placed in direct contact with the skin. The interventional protocol consisted of a session of 40-minute MIRE treatment, three times per week for 2 weeks (total 6 treatments). Therefore, MIRE can deliver up to 34.7 milliwatts/cm^2^ in a 40-minute therapy session [1, 5]. EPN mapping was performed by an investigator, who was blinded to the intervention (MIRE versus sham MIRE). After EPN mapping, the incision in the skin was sutured and the rabbit was kept alive in the same comfortable environment.

### 2.5. Data Analyses

For each MTrS, the prevalence of EPN was defined as the percentage of total occurrences of EPN among 64 searched sites. The mean and standard deviation of the prevalence of EPN within each MTrS in either side before MIRE, immediately after MIRE, and one week after MIRE were calculated. The percent change in value after treatment, compared with the pretreatment value using the formula: % changes = (posttreatment value − pretreatment value)/(pretreatment value) × 100%, was used for statistical analysis. Wilcoxon signed rank test was used to compare the values between the control and experimental sides. Wilcoxon signed rank test of variance was also used to compare the values before MIRE intervention, immediately after MIRE intervention, and one week after MIRE intervention. A *p* value of less than 0.05 was considered statistically significant.

## 3. Results

**Figure 3:**
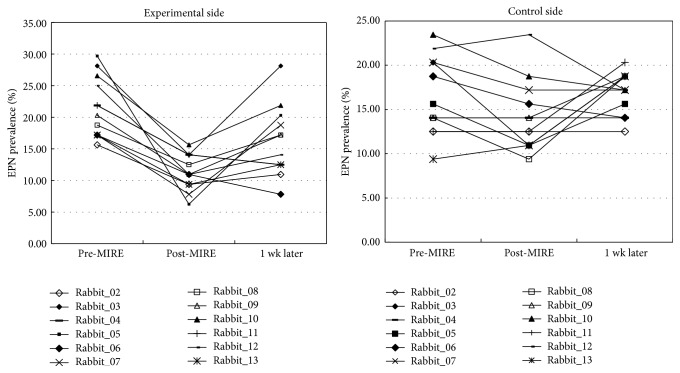
The prevalence of EPN in an MTrS in the experimental side and the control side before MIRE, immediately after MIRE, one week after MIRE. EPN: endplate noise; MIRE: monochromatic infrared photo energy.

In the experimental side, the prevalence of EPN was obviously decreased immediately after MIRE application in every rabbit and then was increased one week later except for rabbits #6 and #13 (Figure 3). On the other hand, the changes of the prevalence of EPN in the control side immediately after the sham treatment and one week later were not so discernible (Figure 3).

Immediately after 2 weeks of MIRE application, the mean prevalence of EPN was significantly decreased compared to that before MIRE application in the experimental side (*p* = 0.0005, Table 1). However, no significant difference was noted in the control side (*p* = 0.2891, Table 1). Between-group comparison showed a significantly higher reduction in the experimental side than that in the control side (*p* = 0.0010, Table 1).

The lasting effect of MIRE, as indicated by the measurement taken one week after the completion of 2-week MIRE treatment, was also investigated. The mean EPN prevalence one week after treatment was significantly lower than that before treatment in the experimental side (*p* = 0.0029; Table 1), but not in the control side (*p* = 0.4697). However, between-group comparison showed that the change in EPN prevalence in the experimental side (−52.29 ± 71.96%) was not significantly different from that in the control side (−28.05 ± 41.01%; *p* = 0.4697, Table 1). This indicated that the lasting therapeutic effect of MIRE after one week of its treatment was weak.

## 4. Discussion

Our study revealed that the EPN prevalence in an MTrS in the rabbit skeletal muscle could be significantly suppressed by the application of MIRE. In a recent human study of the MTrP in the upper trapezius, Kuan et al. [15] found that there was high correlation (*r* = 0.742) between the prevalence of EPN and the pain intensity in the MTrP region, and inversely high correlation (*r* = −0.716) between the prevalence of EPN and the pressure pain threshold. They concluded that the prevalence of EPN in an MTrP region has been shown to be highly correlated with the irritability of that MTrP [15]. Therefore, the decrease in EPN prevalence in an MTrS could be equated to the suppression of the irritability of an MTrP, which would support our hypothesis that MIRE is effective for the management of MPS.

Application of MIRE with 890 nm radiation is supported to increase the localized release of nitric oxide (NO) from hemoglobin [1, 17]. After being released from nitrosothiols in hemoglobin or from endothelial cells, NO will diffuse into smooth muscle cells that line small arteries, veins, and lymphatics [18]. Once being inside the smooth muscle cell, NO will activate guanylate cyclase (GC), which enables the cleavage of two phosphate groups from guanosine triphosphate (GTP). This will result in the formation of cyclic guanosine monophosphate (cGMP), which is important to the phosphorylation of myosin. With myosin being phosphorylated, smooth muscle cell myosin will relax, and the vessels will dilate [19, 20]. NO is a potent endogenous vasodilator. Through the increased release of NO, MIRE has been reported to enhance microcirculation, which will result in “better blood flow, acute delivery of growth factors and white blood cells, fibroblastic differentiation and proliferation, angiogenesis, reduced edema, and mediation of pain” [1, 21, 22].

According to the current literature, most studies on MIRE focused on the restoration of sensation in patients with diabetic peripheral neuropathy with mixed results [2–4, 23]. Many of these studies also lack a more robust study design. For example, one study examined the effect of MIRE in 49 patients with diabetic peripheral neuropathy but did not utilize a control group [3]. Another study of 38 elderly patients evaluated the combined effect of MIRE and physical therapy; the therapeutic benefits from MIRE alone could not be addressed [4]. In addition, the evidence for MIRE treatment for MPS is still lacking. To our knowledge, this experiment is the first placebo-controlled study of the therapeutic effect of MIRE on MTrS (MTrP).

Many clinical signs had been proposed for the diagnosis of MPS [10, 24]. However, controversies still existed regarding their reliability and validity [25]. Simons recommended that “spot tenderness,” “pain recognition,” and “taut band” were the most reliable signs and the minimal criteria for identifying an MTrP, while “referred pain” and “local twitch response” were most useful as confirmatory signs of the MTrP [14]. In our animal study, we used “pain recognition” (animal expressed pain), “taut band,” and R-LTR to confirm the location of MTrS. This approach increased the reliability of our findings.

**Figure 4:**
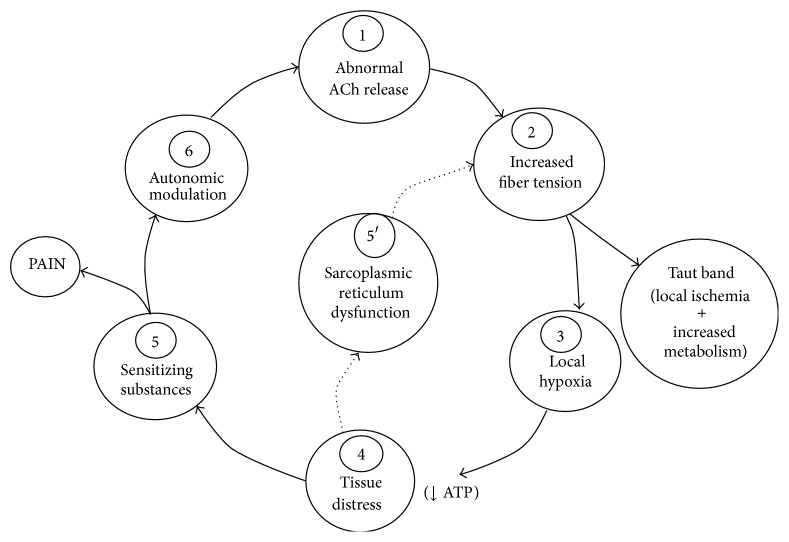
Integrated hypothesis for the pathogenesis of an MTrP: a 6-step positive-feedback cycle (redrawn from Simons, 2004 [26]).

Travell and Simons proposed the “integrated hypothesis of energy crisis,” which has been regarded as today's most accepted theory for the pathogenesis of an MTrP [9, 10]. According to the hypothesis, an initial muscular overload, either from acute trauma, repetitive overuse, or chronic malpositioning, leads to a dysfunctional motor endplate. This results in an excessive release of acetylcholine causing uncontrolled muscle fiber shortening (taut band) and sustained contraction of sarcomeres (contraction knot). Local circulation will thus be impaired even though the metabolic demand is increased. Local hypoxemia and energy crisis are then developed and neurovasoactive substances and neurotransmitters from free nerve endings are also released. In the end, a vicious cycle of energy crisis is formed [9, 10]. In a review article, Simons [26] further substantiated this hypothesis into a 6-step positive-feedback cycle for the pathogenesis of an MTrP (Figure 4). Using a microanalytical technique, Shah et al. [27] measured various biochemicals in the MTrP region in the upper trapezius muscle. These pain and inflammation-associated biochemicals were significantly increased in active patients (those with neck pain and MTrP) than in latent patients (those without neck pain but with MTrP) and normal subjects (no neck pain, no MTrP). Shah's findings strongly supported Simons' integrated hypothesis of energy crisis. With its therapeutic effect of vasodilatation, MIRE shows it could effectively suppress the irritability of the MTrS in the rabbit, which meant MIRE could improve local circulation and ameliorate the energy crisis in the MTrS region, this further supporting Simon's integrated hypothesis of energy crisis for the MTrP.

There have been many therapeutic strategies for the management of MPS, including physical modalities (e.g., thermotherapy and electrotherapy, etc.), manual therapy (e.g., spray and stretch and trigger point pressure release, etc.), MTrP injection (e.g., dry needling, local anesthetics, etc.), and medication (e.g., NSAID, muscle relaxants, etc.) [28]. Most of these therapies have been claimed effective, although none has produced consistent, conclusive evidence. A successful MPS treatment program should target the underlying pathologies and the perpetuating factors for an MTrP. Previously, we reported that botulinum toxin type A, a drug capable of blocking the release of acetylcholine from neuromuscular junction, effectively suppressed the prevalence of EPN in the MTrSs of the rabbit [29]. Botulinum toxin type A has now been shown to be an effective therapeutic option for MPS. In this study, we reported the short-term effectiveness of MIRE in decreasing the EPN prevalence in the rabbit MTrS. However, the therapeutic effect did not significantly last for more than one week. The long-term efficacy of MIRE needs to be further investigated, and combining MIRE treatment with other modalities addressing the etiology of MTrS might be a promising therapeutic strategy.

## 5. Conclusion

The application of MIRE on the MTrS in the rabbit was shown to significantly reduce the prevalence of EPN in the MTrS immediately after treatment. However, the suppressing effect of EPN prevalence would not last for one week after the completion of MIRE treatment. MIRE could effectively suppress the irritability of the MTrS in the rabbit. Our findings in this study suggest that MIRE may be a useful therapeutic option for the management of the MTrP in humans.

## Figures and Tables

**Table 1:** Comparison of EPN prevalence between experimental and control sides (between-group comparison) and among 3 conditions in each side (within-group comparison: before, immediately after, and one week after MIRE application).

	Before MIRE	Immediate after MIRE		One week after MIRE		*p* value^a^		
	Pre (%)	Im (%)	Diff_im_ (%)	1 wk (%)	Diff_1 wk_ (%)	Pre *versus* Im	Pre *versus* 1 wk	Im *versus* 1 wk
Experimental side (*n* = 12)	21.61 ± 4.75	11.33 ± 2.84	46.14 ± 12.81	16.15 ± 5.59	−52.29 ± 71.96	0.0005	0.0107	0.0029
Control side (*n* = 12)	16.41 ± 4.39	14.19 ± 4.02	11.48 ± 18.60	16.93 ± 2.39	−28.05 ± 41.01	0.2891	0.0547	0.4063
*p* value^b^			0.0010		0.4697			

EPN: endplate noise; MIRE: monochromatic infrared photo energy; Pre: % EPN prevalence before MIRE; Im: % EPN prevalence immediately after MIRE; 1 wk: % EPN prevalence one week after MIRE; Diff_im_: % difference between values before MIRE and values immediately after MIRE; Diff_1 wk_: % difference between values before MIRE and values one week after MIRE. Values are mean ± SD or *p* value.

^a^Wilcoxon signed rank test of variance was used to compare the values before MIRE, values immediately after MIRE, and values one week after MIRE.

^b^Wilcoxon signed rank test was used to compare the values between the experimental side and the control side.
